# Antibacterial mechanisms identified through structural systems pharmacology

**DOI:** 10.1186/1752-0509-7-102

**Published:** 2013-10-10

**Authors:** Roger L Chang, Lei Xie, Philip E Bourne, Bernhard O Palsson

**Affiliations:** 1 Department of Systems Biology, Harvard Medical School, Boston, MA 02115, USA; 2 Department of Computer Science, Hunter College, New York, NY 10065, USA; 3 The Graduate Center, The City University of New York, New York, NY 10065, USA; 4 Skaggs School of Pharmacy and Pharmaceutical Sciences, University of California San Diego, La Jolla, CA USA; 5 San Diego Supercomputer Center, University of California San Diego, La Jolla, CA USA; 6 Department of Bioengineering, University of California San Diego, La Jolla, CA 92093-0412, USA

**Keywords:** Structural systems pharmacology, Antibacterial, Metabolic model, Ligand binding, Escherichia coli

## Abstract

**Background:**

The growing discipline of structural systems pharmacology is applied prospectively in this study to predict pharmacological outcomes of antibacterial compounds in *Escherichia coli* K12. This work builds upon previously established methods for structural prediction of ligand binding pockets on protein molecules and utilizes and expands upon the previously developed genome scale model of metabolism integrated with protein structures (GEM-PRO) for *E. coli*, structurally accounting for protein complexes. Carefully selected case studies are demonstrated to display the potential for this structural systems pharmacology framework in discovery and development of antibacterial compounds.

**Results:**

The prediction framework for antibacterial activity of compounds was validated for a control set of well-studied compounds, recapitulating experimentally-determined protein binding interactions and deleterious growth phenotypes resulting from these interactions. The antibacterial activity of fosfomycin, sulfathiazole, and trimethoprim were accurately predicted, and as a negative control glucose was found to have no predicted antibacterial activity. Previously uncharacterized mechanisms of action were predicted for compounds with known antibacterial properties, including (1-hydroxyheptane-1,1-diyl)bis(phosphonic acid) and cholesteryl oleate. Five candidate inhibitors were predicted for a desirable target protein without any known inhibitors, tryptophan synthase β subunit (TrpB). In addition to the predictions presented, this effort also included significant expansion of the previously developed GEM-PRO to account for physiological assemblies of protein complex structures with activities included in the *E. coli* K12 metabolic network.

**Conclusions:**

The structural systems pharmacology framework presented in this study was shown to be effective in the prediction of molecular mechanisms of antibacterial compounds. The study provides a promising proof of principle for such an approach to antibacterial development and raises specific molecular and systemic hypotheses about antibacterials that are amenable to experimental testing. This framework, and perhaps also the specific predictions of antibacterials, is extensible to developing antibacterial treatments for pathogenic *E. coli* and other bacterial pathogens.

## Background

Structural systems pharmacology [1] is the study of drug action through characterization of proteome-wide drug-target interactions and their systemic consequences. A previously developed local structure homology-based approach to predicting ligand binding pockets (SMAP) [2-4] has been applied efficaciously in multiple contexts to study pharmacological phenomena [5-8]. The recent development of a structural biology resource with which to study physiological stresses upon the proteome of *Escherichia coli* K12 MG1655 metabolism [9] has enabled a diversity of potential applications. Thus, we applied the SMAP methodology and the *E. coli* metabolic genome-scale model integrated with protein structures (GEM-PRO), to analyze and predict antibacterial effects of chemical compounds. *E. coli* K12, although not pathogenic under normal circumstances, is a well-characterized laboratory model for enteropathogenic bacteria that infect humans. Thus methods, and perhaps even some specific predictions of antibacterial properties made in this study, are extensible to pathogenic *E. coli* and other bacterial pathogens. In addition to the integrative framework presented in this study for structural systems pharmacology, this effort also included significant expansion of the previously developed GEM-PRO to account for physiological assemblies of protein complex structures with activities accounted for in the *E. coli* K12 metabolic network *i*JO1366 [10]. Results from this study show promising proof of principle for such an analysis framework and raise specific molecular and systemic hypothesis about antibacterials that are amenable to experimental testing.

## Results

### Expansion of GEM-PRO to include protein complexes

**Figure 1:**
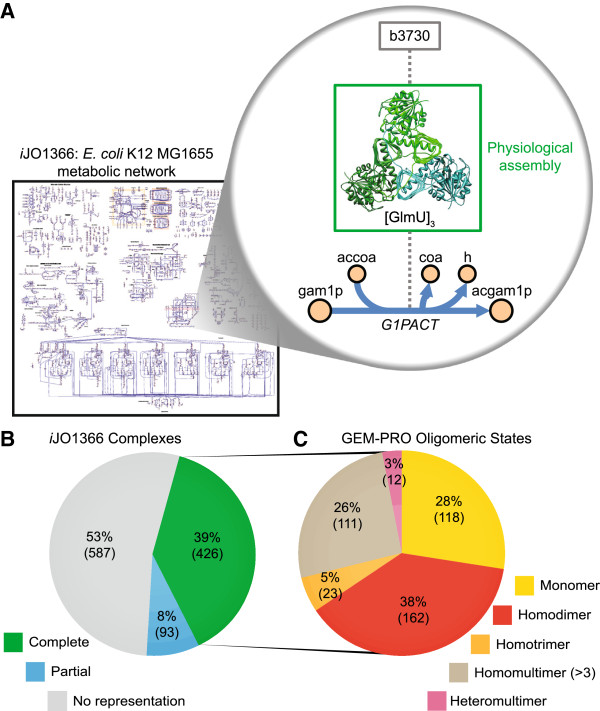
**Complex expansion of *E. coli *GEM-PRO. (A)** This expansion of the E. coli GEM-PRO provides structural coverage of protein complexes included in iJO1366. An example is depicted for the GlmU protein catalyzing the G1PACT reaction. **(B)** Complete and partial coverage of each protein complex by at least one structure is categorized. **(C)** The oligomeric states of complexes for which there is complete coverage in this GEM-PRO are distributed across monomers, homomultimers, and heteromultimers.

Many proteins do not act as monomers in the cell but as part of multimeric protein complexes that may include proteins encoded by one or several distinct genes. The previously constructed *Escherichia coli* genome-scale model integrated with protein structures (GEM-PRO) [9] considered proteins solely as single-peptide chains. As a result, we sought to expand the scope of GEM-PRO to account for the structure of protein complexes. The structures of protein complexes are complementary to the existing single-peptide chain structures already included in the *E. coli* GEM-PRO. The objective was to best represent the physiological assemblies of metabolic enzyme complexes, that is, the best structural representation of the active form of enzyme complexes *in vivo*. A conceptual representation of this expansion with respect to the example reaction of glucosamine-1-phosphate N-acetyltransferase (G1PACT) is displayed in Figure 1A; in this case, the physiologically active form of the GlmU enzyme is a homotrimer.

There are 1106 functional enzymatic complexes [11] known to form among the proteins accounted for in *i*JO1366 [10]. The overall coverage of complexes in this GEM-PRO is 519 out of the 1106 known complexes (Figure 1B); Of these 519 complexes, 426 are completely represented with accurate subunit stoichiometry by a single structure in the expanded GEM-PRO, and another 93 complexes are partially represented by structures, which may not include all distinct polypeptide subunits of the complex or may have incomplete subunit stoichiometry. This effort yielded 527 individual protein structure files, 149 of which were redundant with structures contained in the previously developed GEM-PRO [9]. As is clear from Figure 1B, a slight majority of known complexes are not represented at all in the complex expansion to the GEM-PRO. A combination of the EcoCyc database [11], PDB structure curation [12], computational assessment of symmetry operations on the asymmetric unit of protein crystals [13], and literature review were used to identify a consensus for the most physiologically accurate assemblies currently possible (see Additional file 1: Table S1). These assemblies were distributed among different classes of oligomeric states: monomers, homomultimers, and heteromultimers (Figure 1C). The monomers directly overlap with contents previously reconstructed [9].

### Structure-based prediction of protein targets of antibacterials

**Figure 2:**
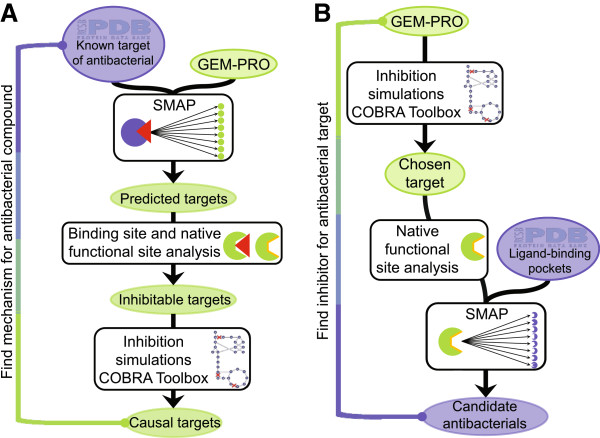
**Antibacterial prediction pipelines. (A)** Screening causal targets for antibacterial activity of input compounds. Seeded with at least one structure of the compound of interest bound to a known target and the GEM-PRO to represent the functional proteome, SMAP is run to predict binding partners within the GEM-PRO. The potential for these predicted binding events to inhibit protein activity is then evaluated based on binding site overlap with native functional sites annotated in the GEM-PRO. Targets exhibiting overlap of antibacterial binding sites and functional sites are then evaluated for their inhibition growth phenotype in the GEM-PRO using the COBRA Toolbox. The inhibitable protein targets leading to deleterious growth phenotypes comprise predictions of causal targets for antibacterial activity. **(B)** Screening inhibitors of desired antibacterial target protein(s). Seeded with the GEM-PRO, metabolic simulations may be performed using the COBRA Toolbox to predict phenotypic impacts of protein inhibition to identify potential antibacterial target protein(s); alternatively, desirable targets may be chosen based on experimental results, such as gene-knockout phenotypes. To search for inhibitors of the chosen targets, the native functional sites of the proteins are identified, as in the GEM-PRO, and passed to SMAP to screen ligand-binding pockets of structures included in the PDB, searching for significant local structural matches. Significant matches comprise potential inhibitors of the chosen target proteins, expected to hold antibacterial properties.

The expanded *E. coli* GEM-PRO was employed prospectively to explore possible currently unknown antibacterial properties. Two pipelines were established to screen for different types of antibacterial associations (Figure 2). Protein targets for antibacterials with unknown mechanisms of action, compounds known to have antibacterial effects but without known molecular targets, were predicted (Figure 2A), and anti-metabolite compounds were also predicted as novel antibacterials to target orphan protein targets without known inhibitors (Figure 2B). Protein-ligand targeting was predicted using the previously developed SMAP method [4]. Some negative and positive control antibacterial compounds were also screened, for which there is existing data on antibacterial properties and established mechanisms of action within metabolism.

A subset of the results of these screens are summarized in Table 1, including novel predicted compound targets and those that displayed antibacterial properties through simulation of inhibition in the metabolic model (described later); the full set of SMAP predictions is presented in Additional file 2: Table S2.

**Table 1:** **Summary of *in silico *antibacterial predictions**

**Screen**	**Ligand ID**	**Target name**	**SMAP prediction (significant)**	**Antibacterial simulation**	**Functional site overlap**
Negative control	BGC	-	-	-	-
Positive control: PEP analogue	FCN	BtuC	×	×	-
Positive control: sulfonamide	YTZ	FolP	×	×	-
Positve control: trimethoprim	TOP	RibD	×	×	×
Positve control: trimethoprim	TOP	IspU	×	×	×
Positve control: trimethoprim	TOP	EntA	×	×	×
Positve control: trimethoprim	TOP	FabG	×	×	×
Positve control: trimethoprim	TOP	KdtA	×	×	-
Positve control: trimethoprim	TOP	MurJ	×	×	-
Positve control: trimethoprim	TOP	WaaB	×	×	-
Positve control: trimethoprim	TOP	MenH	×	×	-
Positve control: trimethoprim	TOP	WaaQ	×	×	-
Positve control: trimethoprim	TOP	MoeA	×	×	-
Positve control: trimethoprim	TOP	TyrA	×	×	-
Positve control: chlorophenol	H3P	-	-	-	-
Antibacterials of unknown mechanism	028	IspA	×	×	×
Antibacterials of unknown mechanism	028	IspB	×	×	×
Antibacterials of unknown mechanism	4AZ	-	-	-	-
Antibacterials of unknown mechanism	2OB	PheA	×	×	×
Antibacterials of unknown mechanism	2OB	AcpP	×	×	×
Antibacterials of unknown mechanism	2OB	EntA	×	×	×
Antibacterials of unknown mechanism	2OB	AtpB	×	×	×
Antibacterials of unknown mechanism	2OB	CyoB	×	×	×
Antibacterials of unknown mechanism	2OB	Cytochrome *bo* terminal oxidase	×	×	×
Antibacterials of unknown mechanism	2OB	Succinate dehydrogenase	×	×	×
Antibacterials of unknown mechanism	2OB	MurJ	×	×	-
Antibacterials of unknown mechanism	2OB	ProC	×	×	-
Antibacterials of unknown mechanism	2OB	ArgA	×	×	-
Antibacterials of unknown mechanism	2OB	IspU	×	×	-
Antibacterials of unknown mechanism	2OB	NuoB	×	×	-
Antibacterials of unknown mechanism	2OB	CyoC	×	×	-
Antibacterials of unknown mechanism	2OB	GdhA	×	×	-
Antibacterials of unknown mechanism	2OB	PpK	×	×	-
Antibacterials of unknown mechanism	2OB	FadE	×	×	-
Novel target: TrpB	F6F	TrpB	×	×	×
Novel target: TrpB	PLT	TrpB	×	×	×
Novel target: TrpB	7MN	TrpB	×	×	×
Novel target: TrpB	IDM	TrpB	×	×	×
Novel target: TrpB	PLS	TrpB	×	×	×
Novel target: PdxB	-	PdxB	-	×	-
Novel target: PyrE	-	PyrE	-	×	-

In the negative control screen for glucose (BGC) SMAP predicted that glucose significantly binds to 7 individual metabolic *E. coli* proteins and 2 protein complexes, one of which is a known target (MglB). Using less stringent significance criteria for the SMAP p-value revealed a second known target (Glk). Some of these targets are expected because glucose is a known substrate of these proteins. Although SMAP does not predict significant binding of glucose to glycogen phosphorylase (GlgP), for which it is a known inhibitor, this protein does rank 4^th^ of 3234 structures for one screen (p-value = 9.55 × 10^-3^). Because we assume that glucose binding targets are the most extensively characterized of all compounds included in this study, these negative control screens were also used to examine the false positive rate of SMAP predictions of ligand binding. Using stated significance criteria (see methods), 9 false positive and 3207 true negative predictions resulted, corresponding to a false positive rate of 0.0028.

Of the positive antibacterial controls, the top SMAP hit for the sulfonamide 4-amino-N-(1,3-thiazol-2-yl)benzenesulfonamide (YTZ) is the known primary target, dihydropteroate synthase (FolP). Two other positive controls, fosfomycin (FCN) and trimethoprim (TOP), were predicted by SMAP to bind significantly to a number of proteins (Table 1), none of which were known targets, leaving these predictions as unresolved but nevertheless putative targets defining unknown mechanisms leading to an antibacterial effect, described further below. The positive control 2,2′-methanediylbis(3,4,6-trichlorophenol) (H3P) was not predicted by SMAP to significantly bind any proteins; although the known primary target (FabI) was ranked 122^nd^ out of 3233 protein structures. The experimentally-characterized binding mode of H3P co-crystalized with bovine glutamate dehydrogenase (GDH) is as a ring consisting of six H3P molecules [14], each molecule interacting both with the GDH homohexamer and with two other neighboring H3P molecules. This complex binding mode may explain the lower than expected significance of SMAP hits for known H3P targets, as the template for the binding site used for the SMAP screen did not capture the six-molecule ring binding mode.

The antibacterial 4-(aminomethyl)benzoic acid (4AZ), with unknown action mechanism, was not predicted to significantly bind to any metabolic proteins. Intriguingly, the two other antibacterials with unknown mechanisms of action screened in this study, (1-hydroxyheptane-1,1-diyl)bis(phosphonic acid) (028) and cholesteryl oleate (2OB), were both predicted as significant binders by SMAP to multiple metabolic proteins (Table 1), suggesting possible mechanisms for their antibacterial activity.

Of the three screens aiming to identify anti-metabolite inhibitors of known essential genes in *E. coli*, SMAP predicted 5 candidate inhibitors for the tryptophan synthase β subunit (TrpB). Predicted TrpB inhibitors include 2-{[4-(trifluoromethoxy)benzoyl]amino}ethyl dihydrogen phosphate (F6F), [3-hydroxy-2-methyl-5-phosphonooxymethyl-pyridin-4-ylmethyl]-L-ryptophane (PLT), (Z)-N-[(1E)-1-carboxy-2-(2,3-dihydro-1H-indol-1-yl)ethylidene]{3-hydroxy-2-methyl-5-[(phosphonooxy)methyl]pyridin-4(1H)-ylidene}methanaminium (7MN), indoline (IDM), and pyridoxyl-serine-5-monophosphate (PLS). Criteria supporting the potential inhibitors of TrpB are listed in Table 1. SMAP screens for inhibitors of erythronate-4-phosphate dehydrogenase (PdxB) and orotate phosphoribosyltransferase (PyrE) failed to predict any significant candidate inhibitors.

Several other known metabolic targets of the control compounds were not predicted by SMAP. In our preliminary control screens, it was hypothesized that there may exist distinct binding pocket motifs for an individual compound such that using a single protein template to search for other targets may not identify all true targets of a compound. Expanding the number of search templates for a single compound, as was done for BGC, FCN, and TOP, indeed identified more significant known targets, supporting this hypothesis.

**Figure 3:**
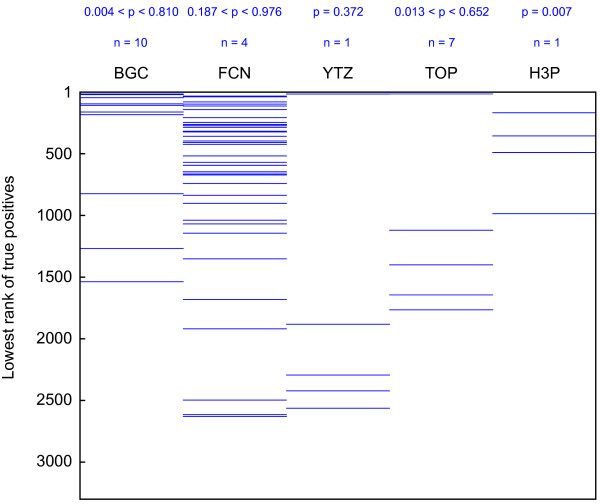
**SMAP performance in recalling true positives.** The lowest rank for each protein structure predicted as an SMAP hit is displayed for the set of known protein targets for the five control compounds. Blue lines indicate the rank position (out of 3237) of a known target for a given compound. n = the number of screens using different protein structure templates performed for each compound. p = the p-value resulting from Mann Whitney statistical tests for individual SMAP results with respect to an individual template screen. BGC: beta-D-glucose; FCN: fosfomycin; YTZ: 4-amino-N-(1,3-thiazol-2-yl)benzenesulfonamide; TOP: trimethoprim; H3P: 2,2′-methanediylbis(3,4,6-trichlorophenol).

To assess the relative accuracy of SMAP in predicting true positive protein-ligand interactions, we performed statistical analysis of the entire set of SMAP results, including insignificant calls. Mann Whitney U-tests were run on the ranked lists of SMAP predictions with respect to each template protein structure, yielding inconsistently statistically significant p-values for some compounds (Figure 3). This result too supports that different binding motifs may exist for an individual compound, as is most apparent for BGC and TOP, which show the widest range of p-values. To highlight the overall efficacy of SMAP in predicting true positives, the results from all screens for a particular compound were combined by considering only the top rank number for each protein structure, whether a known target or not. It is apparent from Figure 3 that the examples BGC, FCN, TOP, and H3P all noticeably support SMAP’s predictive accuracy; however, the stringency of significance criteria used may obscure this ability for many protein-ligand interactions. Because there is no obvious a priori approach to choosing a single structural template for screening a compound that may bind to multiple distinct motifs, our results suggest that using as wide an array of diverse templates as appropriate should be considered when running SMAP screens. This phenomenon may explain some of the false negative SMAP predictions for controls in this study.

### Antibacterial binding site and protein functional site analysis

Next, we utilized the residue-resolution functional annotation of the previously generated *E. coli* GEM-PRO to identify whether the SMAP-predicted ligand binding sites overlapped with known functional sites, such as catalytic and substrate binding sites. Such interactions could be expected to exhibit competitive inhibitory effects. For cases where an SMAP prediction was made on the basis of a protein complex structure, we also identified predicted ligand binding sites at the interface between subunits, which may lead to disruption or prevention of protein complex formation in vivo and therefore have a deleterious impact on enzyme function. Overlap between predicted TOP binding sites and native nucleotide and substrate binding sites occurred on RibD, partial overlap with the catalytic site of IspU, and almost complete overlap with the catalytic sites of both EntA and FabG. The predicted binding sites for 028 completely overlapped with the catalytic site of IspA and overlapped with the substrate binding site and Mg^2+^ ion binding site of IspB. In the case of 2OB, predicted binding sites showed at least partial overlap with the catalytic sites of PheA, CyoB, EntA, AtpB, and AcpP. Predicted 2OB binding sites also had implications with respect to two protein complexes, not exhibited with respect to the complex subunits in isolation. The predicted 2OB binding site on the cytochrome *bo* terminal oxidase appears at the interaction site between CyoB and CyoC. The 2OB binding site also overlapped with the heme binding sites of the SdhC and SdhD subunits of the succinate dehydrogenase complex as well as the protein-protein interaction region between these subunits. These last few predictions speak to the importance of the complex expansion of the GEM-PRO, without which such molecular predictions involving multiple subunit interfaces would not have been possible.

### Simulation of phenotypes from antibacterial target inhibition

Finally, we turned to the metabolic network portion of the *E. coli* GEM-PRO, *i*JO1366 [10], to simulate the outcomes of known and predicted binding events leading to inhibition of protein activity and determine whether or not these events may be detrimental to growth. First, we tested the ability of the model to accurately predict the phenotypic impact caused by inhibition of known targets of all control compounds (Table 2). Inhibition of all known and predicted binding targets of BGC led to no decrease in growth phenotype, accurately predicting the known outcome of the negative control. Inhibition of positive control targets led to no growth or reduced growth rates in the model. In combination, the collective inhibition of all known targets for each positive control compound led to complete growth inhibition, but remarkably, most of these targets individually also led to complete loss of growth if inhibited, only failing to predict deleterious growth phenotypes upon inhibition of FbaA, TolC, and FolA individually.

**Table 2:** Metabolic model performance in predicting antibacterial effects

	**Negative control**			**Positive controls**					
	**BGC**			**FCN**			**YTZ**	**TOP**	**H3P**
No growth upon inhibition	-			AcpP	FabB	FolC	FolP	ThyA	Fabl
				Gmk	IspA	IspB			
				MurA	MurE	PgsA			
				PlsC					
No effect upon inhibition	GalP	GlgY	Glk	FbaA		TolC	FolA	FolA	-
	MglB	XylA	YlaD						

**Figure 4:**
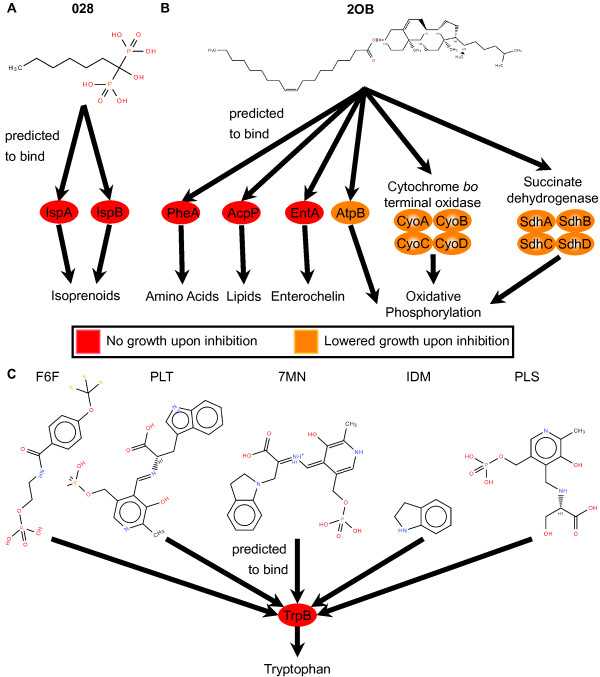
**Predicted antibacterial mechanisms. (A)** Inhibition of predicted binding targets (IspA and IspB) of 028 impacted simulated growth through decreased flux through isoprenoid synthesis pathways, leading to no growth under complete inhibition. **(B)** Through simulated inhibition of predicted binding targets of 2OB, critical metabolic pathways were impacted leading to decreased growth: PheA impacting amino acid synthesis, AcpP impacting lipid synthesis, EntA impacting enterochelin metabolism, and AtpB, cytochrome *bo* terminal oxidase, and succinate dehydrogenase all impacting oxidative phosphorylation. **(C)** Five compounds (F6F, PLT, 7MN, IDM, and PLS) were predicted to bind and competitively inhibit TrpB, leading to decreased tryptophan synthesis.

The effects of inhibition of SMAP-predicted targets were then evaluated in the model. Each of the individual predicted protein targets reported in Table 1 exhibited decreased or no growth upon full inhibition in simulation. These predictions helped to pare down the list of significant SMAP predictions to those that satisfy both lines of evidence for antibacterial effects. With the exception of the FolP-YTZ binding interaction, all of the interactions reported in Table 1 are previously unknown, which suggests that in the case of positive control compounds, we may have uncovered previously unknown antibacterial targets. For the antibacterial compounds with unknown mechanisms of action, we predicted that inhibition of IspA and IspB by 028 leads to decreased growth rate and that inhibition of 14 individual proteins and 2 protein complexes by 2OB leads to decreased growth rate. Further details of the specific pathways impacted by these inhibitory activities were investigated in the flux balance model. The mechanistic models of antibacterial activity of 028, 2OB, and potential inhibitors of TrpB are summarized in Figure 4, with more detailed network flux maps provided in Additional file 3: Figure S1. In the mechanistic model for 028 (Figure 4A), IspA and IspB are inhibited leading to decreased isoprenoid synthesis activity and ultimately no model growth. The mechanistic model for 2OB (Figure 4B) includes inhibition of several proteins (PheA, AcpP, EntA, and AtpB) and protein complexes (cytochrome *bo* terminal oxidase and succinate dehydrogenase) participating in a variety of metabolic pathways (amino acid synthesis, lipid synthesis, enterochelin metabolism, and oxidative phosphorylation) ultimately leading to no model growth.

We also tested if inhibition of the individual protein targets predicted by gene-knockout phenotypes to be effective antibacterial targets leads to growth deficits in the model and found that all three individual inhibitions lead to no growth in the model (Table 1). However, as previously mentioned, our SMAP screens only predicted potential inhibitors of TrpB. The mechanistic model for antibacterial activity of these compounds is presented in Figure 4B, where any of F6F, PLT, 7MN, IDM, or PLS are expected to inhibit TrpB activity, thereby inhibiting tryptophan synthesis and leading to no growth in simulation.

## Discussion

In this study, we have demonstrated the first structural systems pharmacology antibacterial screens for the model bacterium *E. coli*. This effort was enabled in part through the expansion of the *E. coli* GEM-PRO to include protein complexes. In this attempt at reconstruction of metabolic protein complexes, we chose to utilize only those structures supported by strong experimental evidence; however, the scope of this reconstruction could be further expanded through modeling of protein complex structures, as has been attempted by others recently [15]. Our previous and current efforts at reconstructing the *E. coli* metabolic GEM-PRO have enabled *in silico* exploration of diverse forms of physicochemical stress, but much broader expansions are likely to emerge and enable still more diverse avenues of investigation.

One important lesson learned from this study is that availability of only a few static structures to represent proteins may limit the sensitivity of ligand binding prediction. Prospectively, molecular dynamics simulations could be used to generate ensembles of structures [16] for each protein to perhaps include the conformations necessary to uncover more binding interactions, lending greater sensitivity to the prediction approach. Generating such ensembles for the proteins included in this study would be a substantial effort given the high number of protein structures included in this GEM-PRO and the long simulation time scales necessary to model the large conformational changes often important for ligand binding [17]. The expected resultant increase in query database size would also dramatically increase SMAP runtime. Nevertheless, such an undertaking would likely provide a very useful extension of the GEM-PRO as a resource for such screens.

The limiting step of the overall approach is the SMAP runtime, which if implemented on a similar computing resource to that used in this study (see methods) would be limited to the order of hundreds of compounds screened against the *E. coli* GEM-PRO or tens of protein inhibitor screens against the ligand-bound PDB structures. Therefore, orders-of-magnitude more powerful computing resources would be necessary for massively parallel screens.

This study builds upon previous examples [6,9,15,18-20] illustrating how structural and systems biology may combine to have an effect greater than they are capable of in isolation. For example, some of the SMAP predictions of lesser quantitative significance showed promise as antibacterial targets in simulation, sometimes accounting for known antibacterial targets that otherwise would have been called as false negatives by SMAP alone. Conversely, although metabolic model predictions have previously been shown to accurately predict the effects of many targeted gene knockouts [10] and have been applied to select individual and multiple antibacterial targets [21,22], these metabolic models have not yet been capable of pairing these targets with compounds. Not only does the expansion from the GEM to GEM-PRO framework enable prediction of candidate compounds, it enables prediction of specific molecular mechanisms (e.g., competitive inhibition or complex disruption) that explain how the candidate compounds may affect the function of their targets.

In addition to providing a promising proof of principle that such a structural systems biology strategy can be used to understand antibacterial mechanisms, we have made specific predictions of chemical inhibitors of a protein currently unutilized for antibacterial applications (TrpB) and previously unknown mechanisms of existing antibacterial compounds, both those with and without established mechanisms. These predictions represent experimentally testable hypotheses and were generated entirely *in silico*. Therefore, Structural systems pharmacology may seed rapid discovery in the area of antibacterials.

## Conclusions

In this study, we developed an approach that can be used to predict and characterize antibacterial mechanisms either 1) by proteome-wide ligand binding target prediction and subsequent simulation of the effects of such interactions on growth or 2) by metabolic simulation of lethal protein loss of function and subsequent inhibitor prediction. This *in silico* approach bridges the gap between structural and systems pharmacology, linking molecular interactions with phenotypic outcomes. The GEM-PRO in this study enables proteome-wide binding site prediction specifically for *E. coli* metabolism, covering protein conformations in the physiological context of multimeric complexes including potential binding sites at protein-protein interfaces. This is a foundational resource for antibacterial development for pathogenic *E. coli* and related species. The GEM-PRO was utilized to predict binding sites on protein targets for known antibacterials with unknown mechanisms (028 and 2OB), binding sites on previously uncharacterized targets of well-studied antibacterials (FCN and TOP), and potential inhibitors of TrpB. Furthermore, metabolic model simulations predicted specific essential processes by which these binding interactions would lead to antibacterial effects. These represent experimentally-testable hypotheses, and this study as a whole serves as a useful proof of principle for the structural systems pharmacology analysis of antibacterials.

## Methods

### Complex expansion of the *E. coli* GEM-PRO

Enzyme complexes included in the metabolic network *i*JO1366 [10] were reviewed as annotated in EcoCyc [11]. The annotation from EcoCyc includes protein subunit compositions, which served as a starting point for this reconstruction. The EcoCyc subunit compositions were evaluated from a structural perspective based on biological units of crystal structures in the PDB [12] and through thermodynamic analysis of possible physiological assemblies using the PDBePISA software [13]. The most thermodynamically feasible PISA assembly for each complex, based on computed ΔG of dissociation, was compared to PDB biological units and EcoCyc composition annotation for each complex. In many cases, these three sources were in perfect agreement, in which case the PDB biological unit was chosen as the structure to represent the physiological assembly of the complex. However, many discrepancies were also found among the compositions assigned by these sources, including protein membership in complexes but missing stoichiometry in EcoCyc. To reconcile these discrepancies, the scientific literature was reviewed to find experimental evidence supporting the correct physiological assembly for a complex. These references reported data from a variety of experiments including: X-ray crystallography, gel filtration, size-exclusion chromatography, ultracentrifugation, functional assays, substrate binding assays, cooperative analysis, and mutant studies. A few studies also provided evidence from bioinformatics analysis such as kinetic assembly, molecular docking, and orthology-based inference. The consensus of these experimental results and the three preliminary sources was taken to determine the most likely physiological assembly. If the PDB biological unit agreed with the consensus, that structure was taken as the physiological assembly structure. If not, then the PISA structure that best agreed with the consensus was taken as the physiological assembly. In some cases, no PDB structure or PISA assembly completely accounted for the consensus complex assembly. In such cases, multiple structures were taken to represent as many sub-parts of the physiological complex assembly as possible. This resulted in some overlap with single-peptide chain structures included in the previously developed *E. coli* GEM-PRO.

### SMAP implementation

SMAP was installed and run on a Linux server with 48-core 1.9 GHz Opteron processor. For all results reported in this study, SMAP was run with default numerical parameters. The first SMAP run against a given query database and parameter set takes substantially longer than subsequent runs in order to define possible binding pockets (~55 h for the GEM-PRO and ~629 h for all ligand-bound protein structures in the PDB). Average runtimes for subsequent screens in this study were ~4 h and ~49 h against the GEM-PRO and ligand-bound protein structures in the PDB, respectively.

### Protein-ligand interaction predictions

Different types of SMAP screens were run to answer three different types of questions: 1) positive and negative controls for antibacterials with known effective mechanisms in wild type *E. coli* K12 through known metabolic protein targets; 2) antibacterials known to be effective against *E. coli* K12 but with unknown mechanisms of action, seeking to answer the question of whether those compounds may target metabolic functions; 3) searches for potential novel antibacterials that are competitive inhibitors of metabolic proteins known to hinder growth of *E. coli* K12 if subjected to gene knockout. These are all open-ended questions, and candidate compounds and protein targets to be selected for these purposes are non-obvious. Also because SMAP is a method requiring substantial computational resources, the number of screens that could be performed was limited. For these reasons, filtering the wealth of candidate compounds and targets to choose candidates for the screens was necessary. Therefore, large data sources were filtered to pick most promising candidates to test these three types of questions.

### Selecting antibacterial controls for screen

As of September 24, 2012, there are 12,785 chemically distinct ligand molecules represented in at least one PDB structure. Given that SMAP performs best when starting with a well-defined ligand binding site for the search template, we chose only to use experimentally-determined binding sites for this type of screen. The collection of all known antibacterials and their known targets were collected from KEGG [23], EcoCyc [11], DrugBank [24], and ChEMBL [25], and the overlapping set of these and the PDB ligands found. Antibiotic classifications were derived from KEGG, EcoCyc, and DrugBank. All PDB ligands were clustered by their chemical similarity using their canonical SMILES [26] and the EI-Clustering software [27]. The distance matrix output by EI-Clustering was used to form the clusters by hierarchical clustering and a cutoff of 1.15 was determined such that the classified antibiotics were clustered together and not in the same clusters with antibiotics of other classes. Thus, functionally and chemically distinct groups of antibacterials were identified from which to choose positive controls. All curated data used for compound selection is presented in Additional file 4: Table S3. Positive controls were chosen from these groupings such that they represented a breadth of antibacterial classes and chemical clusters and only if they had at least one known metabolic protein target in *E. coli*.

Glucose was chosen as a negative control for this study due to multiple advantageous properties. Glucose is a molecule well known to cross the *E. coli* cellular membrane and not to exhibit negative effects on growth, as it is a primary carbon source for WT *E. coli*. Therefore, negative phenotypic effects would be completely unexpected in an accurate model. Glucose has many well-characterized binding sites, supported by a high number (> 400) of PDB structures in which it is co-crystalized with diverse proteins (representatives from > 200 protein clusters, with a 50% sequence identity threshold). Known binding targets for glucose in the *E. coli* GEM-PRO include five enzyme catalytic sites for which it is a known substrate and also as a competitive inhibitor of GlgP [28], providing test cases for ligand binding prediction as well as growth phenotype simulation upon target inhibition. As a small molecule (180 Da) within a standard deviation of the mean molecular mass of crystalized ligands in the PDB (376+/−196 Da), glucose is a reasonable representative of characterized ligands in terms of size. Glucose also satisfies Lipinski’s rule of five [29], indicative of its drug-like chemistry. These factors taken together make glucose a good negative control for all steps of our predictive approach.

### Selecting antibacterials with unknown mechanisms of action for screening

The ChEMBL database [25] was reviewed to find biological assays in which antibacterial activity of compounds was identified in *E. coli*. This set of compounds was searched for those with no known binding partners in WT *E. coli* according to KEGG, EcoCyc, DrugBank, ChEMBL, or the PDB. We then prioritized for those compounds that are ligands in PDB structures of only non-bacterial proteins. Small compounds consisting only of C, H, N, O, P, and S elements were chosen from this set as the orphan antibacterials of interest for this study. This data is also contained in Additional file 4: Table S3.

### Selecting orphan protein targets for screening

Previously published essentiality screens and simulations of the *E. coli* K12 single-gene knockout library grown on glucose minimal medium [10] were analyzed to choose novel antibacterial protein targets to search for anti-metabolites to inhibit. Phenotypes with very low growth at the end of the experiment (OD_600_ < 0.26) were selected. Priority was given to proteins without known inhibitors in EcoCyc, DrugBank, or ChEMBL. From this set, three target proteins were chosen that bind to a high number of ligands in the PDB, have a low number of native metabolic substrates as annotated in *i*JO1366, and for which there is structural coverage in the GEM-PRO of the individual proteins, protein complexes, and catalytic sites. The curated data used for orphan protein target selection is presented in Additional file 5: Table S4.

### Prediction of antibacterial targets

In searching for possible metabolic protein targets for known antibacterial compounds, template structures were chosen from PDB crystal structures that included the compound bound to a protein. These structures were used with SMAP to search for potential binding pockets for these antibacterial compounds within both the previously published *E. coli* GEM-PRO and also the newly-generated physiological complex assemblies. The entire set of PDB proteins was clustered using a 50% sequence identity cutoff. The best resolution structure from each cluster that contained the ligand of interest was chosen as an alternative template for SMAP screens. SMAP was used to screen each template in turn across the database of proteins comprising the GEM-PRO structures. SMAP hits were considered significant for a p-value < 1.0 × 10^-4^ and Tanimoto coefficient > 0.5. A secondary tier of lesser significance was determined using just the aforementioned p-value criterion.

### Prediction of anti-metabolite protein inhibitors

Searching for possible inhibitors of predicted antibacterial metabolic protein targets was performed by taking the structure of the protein target of interest from the *E. coli* GEM-PRO, docking [30] the primary native metabolic substrate into the known catalytic site (as annotated in the GEM-PRO), and using the resulting structure as a template for SMAP screens. SMAP was then used to search across all ligand-bound protein structures in the PDB, excluding structures that only bind metal ions or metabolites included in *i*JO1366, to find ligands that bind to structurally similar sites. The query database contained 51,608 PDB structures. SMAP was run specifying that only ligand binding sites be considered. SMAP hits were considered significant with p-value < 1.0 × 10^-4^ and Tanimoto coefficient > 0.5. A secondary tier of lesser significance was determined using just the aforementioned p-value criterion.

### Simulating protein inhibitory effects

The *E. coli* metabolic network *i*JO1366 [10] was loaded into the COBRA toolbox [31] from the published SBML model using Matlab. Since the time of publication of *i*JO1366 a thermodynamic constraint error was discovered in the published model; as a result, the malate oxidase, “MOX,” reaction was set as irreversible. The superoxide dismutase, “SPODM,” reaction was set with an initial upper bound of 1000 as well. The objective function was set as the complete wild type biomass reaction “Ec_biomass_*i*JO1366_WT_53p95M.” Default exchange reaction constraints were used, except for a glucose uptake lower bound of −8 mmol/gDW/h and an oxygen uptake lower bound of −18.5 mmol/gDW/h, representing aerobic growth on glucose. These basic constraints were used for all reported simulations in this study.

The combined sets of known targets and predicted targets were first tested for antibacterial effects by constraining all associated reactions to 0 flux and then maximizing biomass using flux balance analysis (FBA) [32]. Individual targets were tested in the same manner to determine causal targets from the broader sets. Resulting biomass fluxes were compared to a simulated untreated condition where just the basic constraints were imposed and biomass was maximized; any decrease in biomass flux relative to the untreated condition was considered a prediction of antibacterial effect by degree of decrease.

### Analysis of impact of protein-ligand binding on molecular function

The specific amino acid residues comprising the ligand binding sites predicted by SMAP were compared to residue-resolution functional annotation contained in the original GEM-PRO [9]. If precise residues overlapped between these sets, we flagged these proteins as having predicted binding sites for the given ligand that should be seen as competitively inhibitory since they would bind to the same location as substrates required for normal function. Functional features included in this analysis consisted of catalytic sites and substrate binding sites. For SMAP query structures that were protein complexes containing multiple subunits, if the predicted ligand binding site included residues from distinct subunits, we flagged these as possible ligand binding events that could prevent or disrupt complex formation and therefore function.

## Competing interests

The authors declare that they have no competing interests.

## Authors’ contributions

RLC, PEB, and BOP conceived and designed the study. RLC implemented and performed computational and statistical analyses. LX helped implement and interpret results of SMAP. All authors helped to draft the manuscript. All authors read and approved of the final manuscript.

## Supplementary Material

Additional file 1: Table S1 Excel file containing indices providing details about the protein complex structures contained in the GEM-PRO. GEM-PRO file naming convention: (1) The PDB ID is given separated from the concatenated chain IDs by an underscore. (2) The stoichiometric presence of each chain is annotated in parentheses following each chain ID. No parenthetic number indicates a stoichiometry of 1. (3) Suffixes for PDB biological units are retained, such as “.pdb1”, “.pdb2”, or “.pdb3”. (4) The suffixes “.pisa1.pdb”, “.pisa2.pdb”, “.pisa3.pdb”, and “.pisa4.pdb”, indicate that the structure is an output of PDBePISA. The number following “.pisa” indicates the rank within the list of possible structures returned by PDBePISA. Click here for file

Additional file 2: Table S2 Excel file containing summary results from all SMAP screens. Click here for file

Additional file 3: Figure S1 Sub-networks of *i*JO1366 affected by simulated inhibition of predicted targets of 028, 2OB, and TrpB inhibition by F6F, PLT, 7MN, IDM, or PLS. Reactions in green, red, and yellow are those directly affected by predicted target inhibition by 028, 2OB, and one of the predicted TrpB inhibitors, respectively. Reactions with thicker lines represent those with lower magnitude flux upon simulated exposure to these compounds. Colored dashed lines are drawn from each compound to their predicted causal targets following the same color scheme described above. Click here for file

Additional file 4: Table S3 Excel file containing all curated data used to select ligands for antibacterial mechanism screens. Click here for file

Additional file 5: Table S4 Excel file containing all curated data used to select orphan protein targets to screen for inhibitors. Click here for file
