# Supplementary material for: Antibacterial mechanisms identified through structural systems pharmacology
Source: BMC Syst Biol. 2013 Oct 10;7:102. doi: 10.1186/1752-0509-7-102 (PMC3853765; doi:10.1186/1752-0509-7-102)
Supplement: Additional file 3: Figure S1 — Sub-networks of iJO1366 affected by simulated inhibition of predicted targets of 028, 2OB, and TrpB inhibition by F6F, PLT, 7MN, IDM, or PLS. Reactions in green, red, and yellow are those directly affected by predicted target inhibition by 028, 2OB, and one of the predicted TrpB inhibitors, respectively. Reactions with thicker lines represent those with lower magnitude flux upon simulated exposure to these compounds. Colored dashed lines are drawn from each compound to their predicted causal targets following the same color scheme described above. [file 1752-0509-7-102-S3.pdf]

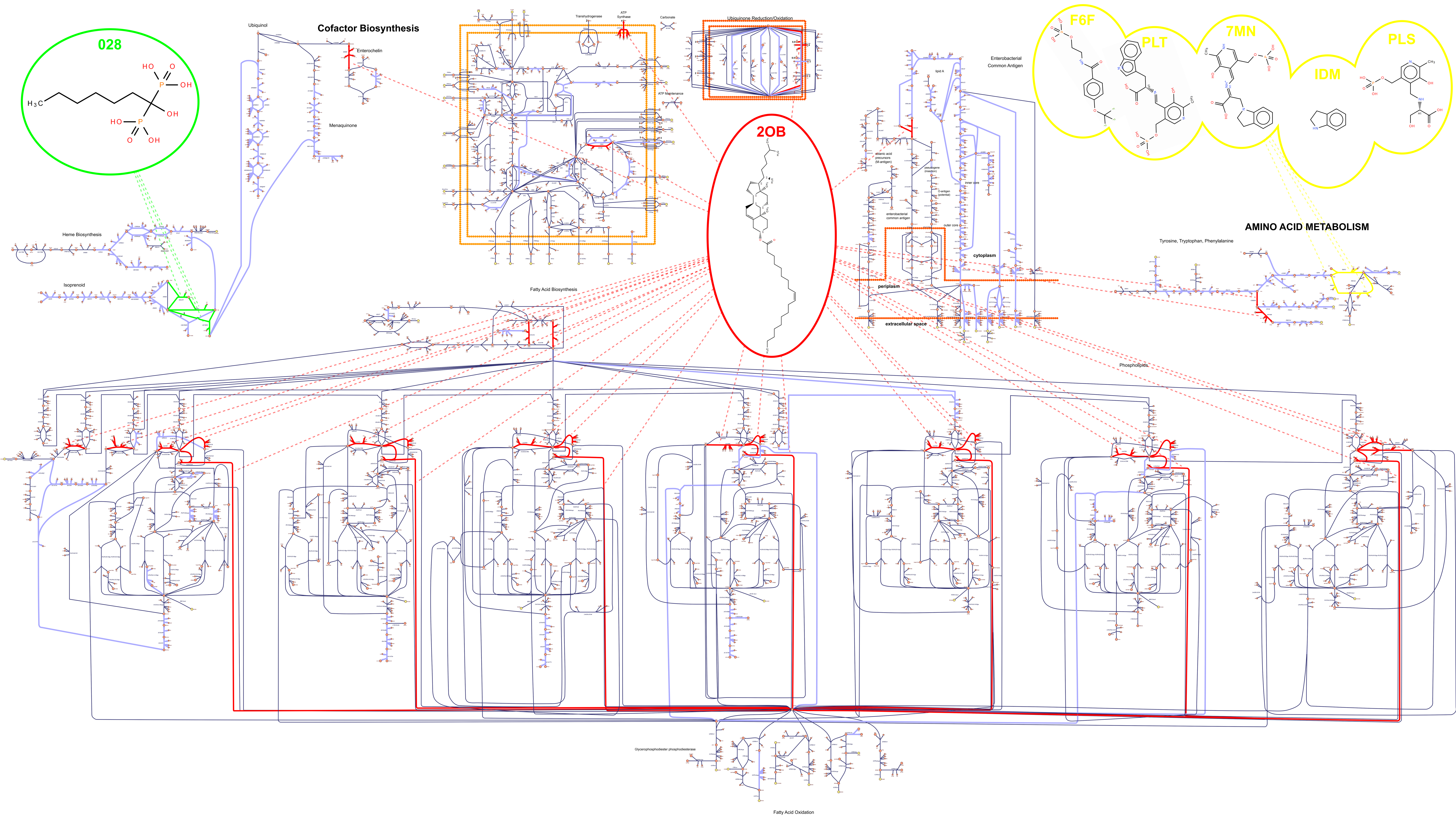
028
Cofactor Biosynthesis
Ubiquinol
Enterochelin
Menaquinone
Heme Biosynthesis
Isoprenoid
Transhydrogenase
ATP Synthase
Carbonate
ATP Maintenance
Ubiquinone Reduction/Oxidation
2OB
Enterobacterial Common Antigen
lipid A
olanic acid precursors (M-antigen)
pseudogene (insertion)
O-antigen (potential)
inner core
enterobacterial common antigen
outer core
cytoplasm
periplasm
extracellular space
F6F
PLT
7MN
IDM
PLS
AMINO ACID METABOLISM
Tyrosine, Tryptophan, Phenylalanine
Fatty Acid Biosynthesis
Phospholipids
Glycerophosphodiester phosphodiesterase
Fatty Acid Oxidation
